# Phosphate and IL-10 concentration as predictors of long-covid in hemodialysis patients: A Brazilian study

**DOI:** 10.3389/fimmu.2022.1006076

**Published:** 2022-09-30

**Authors:** Hugo L. Corrêa, Lysleine A. Deus, Thaís B. Araújo, Andrea L. Reis, Carlos E. N. Amorim, André B. Gadelha, Rafael L. Santos, Fernando S. Honorato, Daisy Motta-Santos, Carmen Tzanno-Martins, Rodrigo V. P. Neves, Thiago S. Rosa

**Affiliations:** ^1^ Department of Physical Education, Catholic University of Brasília, Brasília, DF, Brazil; ^2^ Physical Education Department, Federal University of Maranhão, Maranhão, Brazil; ^3^ Pontifical Catholic University of Minas Gerais and Sports Department, EEFFTO, UFMG, Belo, Horizonte, Brazil; ^4^ Clinical Group Home Dialysis Center and RenalClass, São Paulo, Brazil; ^5^ Serviço de Preparação Física/COPSA/COSAU/DIGEP/SA/SG/Presidência da República, Brasília, Brazil; ^6^ Seção de Educação Física do Colégio Militar de Brasília, Brasília, Brazil

**Keywords:** inflammation, phosphate, post-COVID, nephrology, hemodialysis

## Abstract

**Background:**

The global burden of persistent COVID-19 in hemodialysis (HD) patients is a worrisome scenario worth of investigation for the critical care of chronic kidney disease (CKD). We performed an exploratory *post-hoc* study from the trial U1111-1237-8231 with two specific aims: i) to investigate the prevalence of COVID-19 infection and long COVID symptoms from our Cohort of 178 Brazilians HD patients. ii) to identify whether baseline characteristics should predict long COVID in this sample.

**Methods:**

247 community-dwelling older (>60 years) patients (Men and women) undergoing HD (glomerular filtration rate *<* 15 mL/min/1.73m^2^) with arteriovenous fistula volunteered for this study. All patients presented hypertension and diabetes. Patients were divided in two groups: without long-COVID and with long-COVID. Body composition, handgrip strength, functional performance, iron metabolism, phosphate, and inflammatory profile were assessed. Patients were screened for 11-months after COVID-19 infection. Results were considered significant at *P* < 0.05.

**Results:**

We found that more than 85% of the COVID-19 infected patients presented a severe condition during the infection. In our sample, the mortality rate over 11-month follow was relatively low (8.4%) when compared to worldwide (approximately 36%). Long COVID was highly prevalent in COVID-19 survivors representing more than 80% of all cases. Phosphate and IL-10 were higher in the long COVID group, but only phosphate higher than 5.35 mg/dL appears to present an increased prevalence of long COVID, dyspnea, and fatigue.

**Conclusion:**

There was a high prevalence of COVID-19 infection and long COVID in HD patients from the Brazilian trial ‘U1111-1237-8231’. HD clinics should be aware with phosphate range in HD patients as a possible target for adverse post-COVID events.

## Introduction

Coronavirus disease 2019 (COVID-19), caused by the severe acute respiratory syndrome coronavirus-2 (SARS-CoV-2) has impacted worldwide public health (1, 2). More than 6 million deaths were registered by May 2022, being more evident in Europe and Americas (https://covid19.who.int/; assessed 30 May 2022). Recent studies suggest that are several risk factors for severe or fatal COVID-19 that differs according to pre-existing diseases status (1, 3).

Chronic kidney disease (CKD) has emerged as an independent risk factor for the severeness of COVID-19 illness (1, 3–5). Such adverse condition appears to be related with impaired immune function, low-grade chronic inflammation, cardiovascular diseases, frailty, and endothelial dysfunction, which are enhanced according to the severity of CKD, especially in patients undergoing maintenance hemodialysis (HD) (6–8). The Centre for the Mathematical Modeling of Infectious Diseases COVID-19 working group (1) suggested CKD as an important risk factor for severe COVID-19. Furthermore, the ERA-EDTA Council (European Renal Association and European Dialysis and Transplantation Association) and the ERACODA Working Group demonstrated that HD (2021) (9), organ transplantation, and low renal function (estimated glomerular filtration rate < 30 mL/min/1.73m^2^) are associated with highest mortality risk from COVID-19. Carriazo et al. (7) showed that the increased mortality rate persists during the one-year after COVID-19 diagnosis in HD patients. Therefore, it is recommended that patients undergoing HD that were infected with COVID-19 should be monitored continuously to avoid future complications, especially in the first three months (8).

The long-term of new, returning, or ongoing health issues after the first infection with SARS-CoV-2 is recognized as post-acute COVID-19 syndrome conditions or long COVID (10–12). This scenario can last ranging from weeks to years. Post-COVID symptoms mostly reported are fatigue, fever, shortness of breath, cough, body aches, heart palpitations, neurological symptoms, diarrhea, stomach pain, and body aches (13). Noteworthy, subjects with underlying health conditions, including HD patients are likely to develop long-COVID (8, 11–14). Considering the global interest on how COVID-19 symptoms might persist in HD patients, as well as possible targets to avoid this condition, we performed an exploratory *post-hoc* study from the trial U1111-1237-8231 (15) with two specific aims: i) to characterize COVID-19 infection and long COVID symptoms from our Cohort of 178 Brazilians HD patients. ii) to identify whether baseline characteristics should predict long COVID in this sample. This study provides relevant information regarding the prevalence of COVID-19 and long COVID in HD patients and suggested phosphate and interleukin (IL-10) as possible targets associated with post-COVID conditions.

## Methods

**Figure 1:**
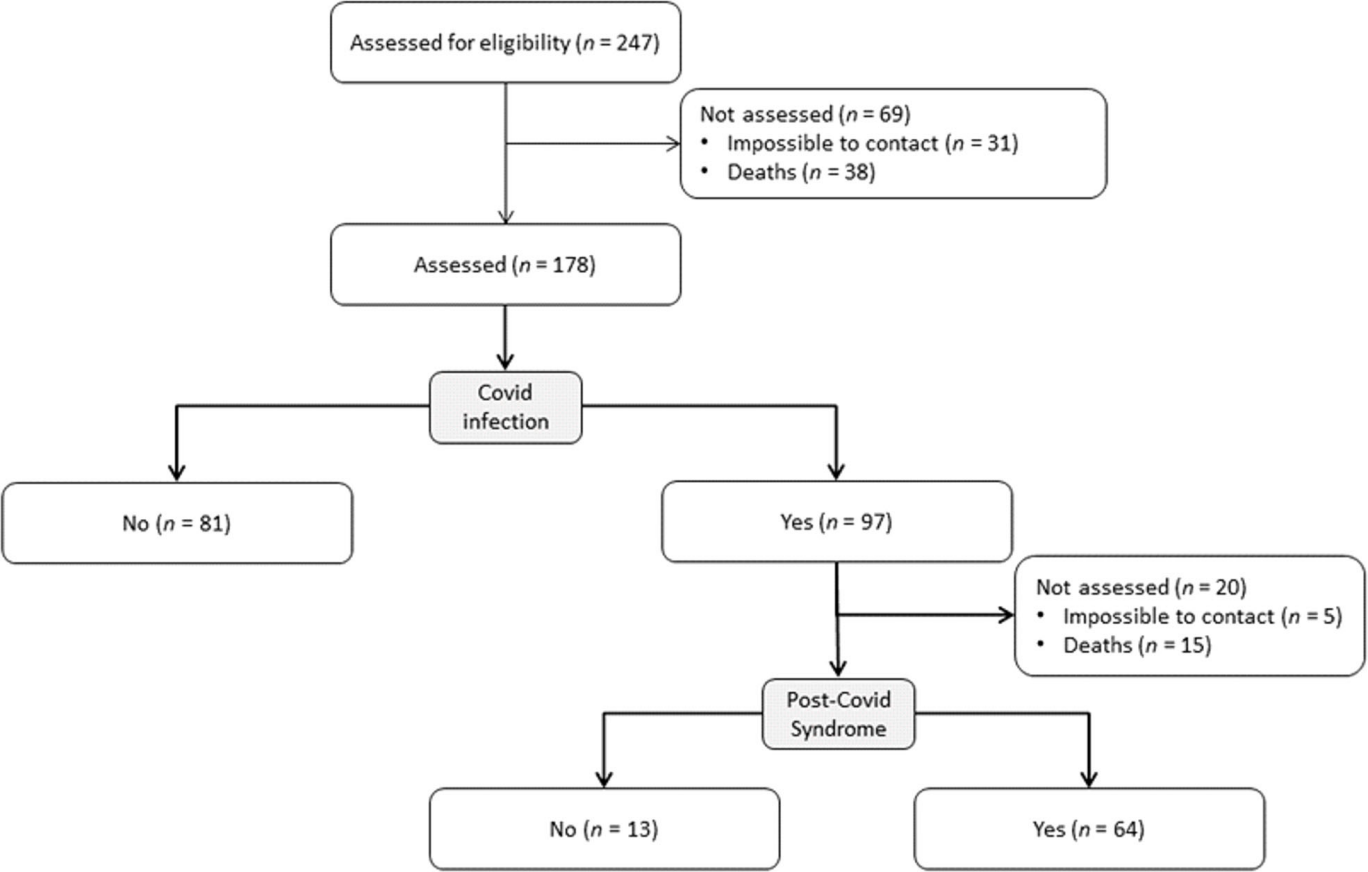
Participant’s flow-chart.

This study is a follow-up study of data from the trial U1111-1237-8231. This study has been conducted prospectively in hemodialysis clinics from Brazil and has been previous described (15). It is worth to state that this manuscript presents additional data from the study of 15. Briefly, this study covered 247 (two hundred and forty-seven) community-dwelling older patients undergoing HD (glomerular filtration rate *<* 15 mL/min/1.73m^2^) with arteriovenous fistula who volunteered for this study. All patients presented hypertension and diabetes. Eligibility criteria for participants were: (i) age equal to or older than 60 years; (ii) hemodialysis for at least 3 months; (iii) dialysis at least three times per week; and (iv) no significant medical complications in the last 3 months, except for vascular access correction. Exclusion criteria were: (i) systemic lupus erythematosus; (ii) congenital kidney malformation or some auto-immune disease that affects the kidneys; (iii) severe decompensated diabetes. Participants were informed about the procedures and possible risks of participation in the study. Before participation in the research project, each participant completed a medical history questionnaire and voluntarily signed a written informed consent form. The study was conducted according to the Declaration of Helsinki (1975). All methods and procedures were approved by the Local Human Research Ethics Committee, Brazil (no 08856012.6.0000.5505 and updated 23007319.0.0000.0029). Furthermore, a new protocol register number was made and approved for conducting the study with COVID-19 patients (no 47011221.2.0000.5086). After 11-month follow-up only 178 patients were analyzed for COVID-19 and long-COVID. All patients’ selection can be found in **Figure 1**.

### Anthropometric assessment

All subjects were weighed on a mechanical scale (Filizola^®^, São Paulo, Brazil), and height was measured with a stadiometer built into the scale (precision: 0.5 cm). Waist circumference was assessed at the level of umbilicus using an anthropometric tape (Sanny^®^, São Paulo, Brazil). Body composition was measured using a Prodigy Advance Plus (LUNAR,Corp/General Electric; Madison, Wisconsin, USA) dual-energy X-ray absorptiometry (DXA) according to previously specified procedures (16). All measurements were carried out by the same experienced researcher.

### Handgrip strength and functional performance

Handgrip strength was measured with a hydraulic hand dynamometer (Jamar^®^ - Sammons Preston, Bolingbrook, USA). According to the recommendations of the American Society of Hand Therapists, measurements were performed with participants in a sitting position, elbow joint at 90°, forearm in a neutral position, and wrist between 0° and 30° of extension. The best performance of three trials in the contralateral arm to the arteriovenous fistula was recorded and used for the present analyses (17, 18). Functional performance was evaluated by the Timed Up-and-Go (TUG) test and six-minute walking test (6MWT). Procedures were fully explained before the assessment, followed by a familiarization attempt as described elsewhere (19).

### Medications in use

All patients had similar medication regimen. Briefly, for glucose control the most common treatment was long-acting insulin. Erythropoietin was administered raging from 50 to 100 UI/kg three times/week with constant adjustments for anemia control. Simvastatin, Pravastatin, Fluvastatin, and Rosuvastatin with the following doses/day: 5 – 20mg, 10 – 40mg, 80mg, 10 – 20mg, respectively. Phosphate binder Sevelamer was administered 800mg three times/day to control phosphate levels between 5.5 and 7.5mg/dL.

### Blood collection

Venous blood samples were obtained from all patients to measure biochemical variables using dry and EDTA containing tubes. All blood was obtained before COVID infection. Samples were obtained in the morning (8 to 12 h of fasting) and all patients were instructed not to practice any physical activity for 48 h before. Samples were centrifuged at 1500 x g for 15 min; after processing, the specimens were aliquoted into cryovials and stored at -80° C.

### Inflammatory profile

The systemic TNF-α, IL-6, and IL-10 levels were measured in triplicate by enzyme-linked immunosorbent assay (ELISA) kits from R&D Systems (Minneapolis, MN, USA) according to the manufacturer’s instructions. The detectable limits for TNF-α, IL-6, and IL-10 were 10, 18, and 0.2 pg,mL^-1^, respectively. The overall intra- and inter-assay CVs for inflammatory markers and hepcidin were in a range from 2.3 to 10%.

### COVID and post-acute COVID-19 syndrome assessment

Covid diagnostic by PCR and severity, including hospitalizations, deaths, nursery, intensive care unit, and supplementary O_2_ was obtained through the 11-months follow-up by checking the medical records from the HD clinic. Long COVID symptoms were recorded by performing a semi-structured telephone call, conducted through all 11-months follow-up. During the interviews patients were asked if they are feeling dyspnea, dizziness, headache, myalgia, cognitive deficits, and fatigue. After that, the interviewer checked with the responsible person for the patient if all the symptoms are corrected recorded and if they were not evident before COVID infection. At least ten contact attempts were made to each volunteer with missed calls. All telephone calls were made by the same person, who had experience with telephone interviews (20). The long-term of new, returning, or ongoing health issues after the first infection with SARS-CoV-2 recognized as post-acute COVID-19 syndrome conditions or long COVID (10–12).

### Statistical analysis

Descriptive characteristics are presented as means and standard deviations unless otherwise noted. The Shapiro–Wilk test and Levene were used to verify data distribution nature and homogeneity, respectively. X^2^ tests were performed to compare categorical variables, while continuous variables were tested for significance by performing a student t test to compare groups (with and without post-covid syndrome. The Receiver Operating Characteristic (ROC) curve was generated plotting sensitivity (y-axis) as a function of 1-specificity (x-axis). Sensitivity or true-positive points to the individual that was correctly diagnosed with the outcome by an indicator. 1-specificity or true-negative refers to the subjects that were wrongly diagnosed with the outcome by an indicator. In the present study, to be considered an indicator, the area under the ROC curve should be higher than 0.50 and lower than 1.00. Therefore, a larger area indicates a greater discriminatory power of the respective indicator. Herein the two elected indicators were phosphate and IL-10. To establish the cut-off point, was considered the closest value between sensitivity and 1-specificity not lower than 0.60. Furthermore, the odds ratio (95% confidence interval [CI]) were obtained for phosphate and IL-10 to verify the likelihood to present post-covid syndrome according to the cut-off points established for both variables.

After performing the aforementioned analysis, phosphate was elected as a possible predictor of the post-covid syndrome. Therefore, the subjects were subgrouped according to the cut-off points established by the ROC curve (group 1: phosphate <5.35; group 2: phosphate >5.35). For that purpose, the groups were than compared using a chi-squared test. Results were considered significant at *P* < 0.05, and all analysis were performed using *IBM Corp. Released 2013. IBM SPSS Statistics for Windows, version 22.0. Armonk, NY: IBM Corp. And GraphPad Prism version 8.0.0 to Windows, GraphPad Software, San Diego, california, EUA, www.graphpad.com “*.

## Results

**Figure 2:**
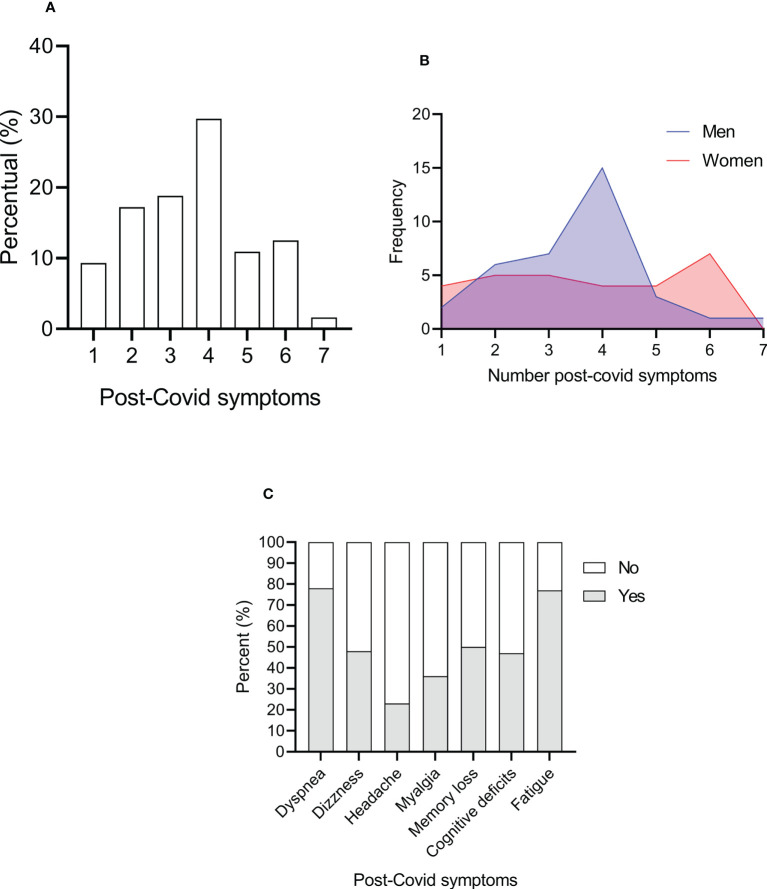
Percent of long covid symptoms **(A)**, frequency of men and women for each number of post-covid symptoms, and frequency of each symptom in hemodialysis patients **(B)**. Percent of each post-covid symptoms **(C)** .

COVID infection occurs raging from April to December of 2020. The prevalence of COVID-19 in our Cohort was 54.5%. 87% were hospitalized, 91.6% needed nursery, 98.8% used supplementary O_2_, and 91.6% were hospitalized in an intensive care unit. Time of hospitalization ranged from 1 to 11 days (mean ± SD: 5.95 ± 3). Furthermore, patients were not vaccinated yet. As observed on **Figure 1**, there were 15 deaths (8.4%) from the patients infected with COVID-19 in an eleven-months follow-up. From the 77 survivors (79.38%), 64 developed long covid (83.12%). Approximately 30% of the patients presented at least four post-covid symptoms, which the most prevalent were dyspnea and fatigue (78 and 77%, respectively). No differences were found between men and women related to post-covid syndrome (*P* > 0.05). See **Figure 2**

Baselines characteristics stratified by the long covid diagnosis are described on **Table 1**. Patients that developed long covid demonstrated higher values of phosphate and IL-10 when compared to patients without long covid. In this context, a ROC curve was generated to verify the cut-off points of the aforementioned variables as possible risk factors for long covid (**Table 2**).

**Table 1:** Baseline characteristics stratified by the presence of long COVID.

Variables	Total (n=64)	Long COVID		*P* Value
		No (n=13)	Yes (n=64)
Age (years)	66.8 ± 3.34	65.31 ± 4.77	67.11 ± 2.99	0.211
Body mass (kg)	75.2 ± 13.91	73.97 ± 18.49	75.82 ± 12.97	0.735
BMI (kg/m2)	26.85 ± 3	26.42 ± 3.78	27.08 ± 2.77	0.826
Waist circumference (cm)	95.66 ± 11.31	96.78 ± 14.77	95.82 ± 10.75	0.353
Fat-free mass (kg)	44.82 ± 5.07	43.23 ± 6.84	45.13 ± 4.55	0.995
Body fat (kg)	30.22 ± 9.48	30.74 ± 12.37	30.71 ± 9.18	0.842
Handgrip (kgf)	24.06 ± 6.45	22.54 ± 6.5	23.86 ± 6.73	0.519
TUG (s)	8.55 ± 2.54	9.12 ± 2.35	8.53 ± 2.47	0.432
6MWT (m)	495.34 ± 102.33	498.08 ± 128.29	491.92 ± 96.91	0.844
Hemoglobin (g/dl)	12.14 ± 1.29	11.48 ± 1.18	12.06 ± 1.41	0.242
Iron (μg/dl)	77.69 ± 20.81	81.28 ± 20.37	74.99 ± 23.65	0.447
Ferritin (ng/ml)	404.79 ± 145.58	389.39 ± 179.46	428.77 ± 151.3	0.484
Hepcidin (ng/mL)	39.76 ± 10.58	42.11 ± 10.64	37.47 ± 10.6	0.225
Phosphate (mg/dl)	5.25 ± 0.91	**4.28 ± 0.67**	**5.26 ± 0.91**	**0.003**
TNF (pg/mL)	16.46 ± 6.38	17.62 ± 6.62	17.35 ± 6.37	0.908
IL 10 (pg/mL)	9.76 ± 3.21	**6.84 ± 1.92**	**9.77 ± 3.13**	**0.007**
IL 6 (pg/mL)	3.24 ± 1	3.55 ± 1.27	3.09 ± 1.03	0.243

Independent Students T test was applied to compare groups. BMI, body mass index; TUG, timed up and go; 6MWT, six-minute walking test; TNF, tumor necrosis factor, IL, interleukin.

Data expressed by mean and standard deviation. Bold values highlight statistical significances.

**Table 2:** Area under the ROC curve and odds ratio in presenting post-covid syndrome.

Variables	AUC ROC	95% CI	Cut-off point	Sensitivity/1-especificity	Odds ratio	95% CI
Phosphate	0.804	0.656 - 0.952	5.35 (mg/dL)	0.900/0.632	11.118	1.279 - 96.661
IL-10	0.795	0.661 - 0.929	9.75 (pg/mL)	0.900/0.600	7.286	0.838 - 63.345

The cut-off point stablished by the ROC curve for phosphate and IL-10 was 5.35 mg/dL and 9.75 pg/mL, respectively. Both variables seem to predict long covid in maintenance HD patients (area under the ROC curve higher than 0.50 and lower than 1.00). However, only phosphate presented a significant odds ratio (11.118%CI: 1.279 – 96.661). See **Table 2**. Noteworthy, IL-10 and phosphate levels from patients who did not survive were higher than the cut points proposed in the study: 11.25 ± 3.73 and 5.63 ± 0.87, respectively.

**Figure 3:**
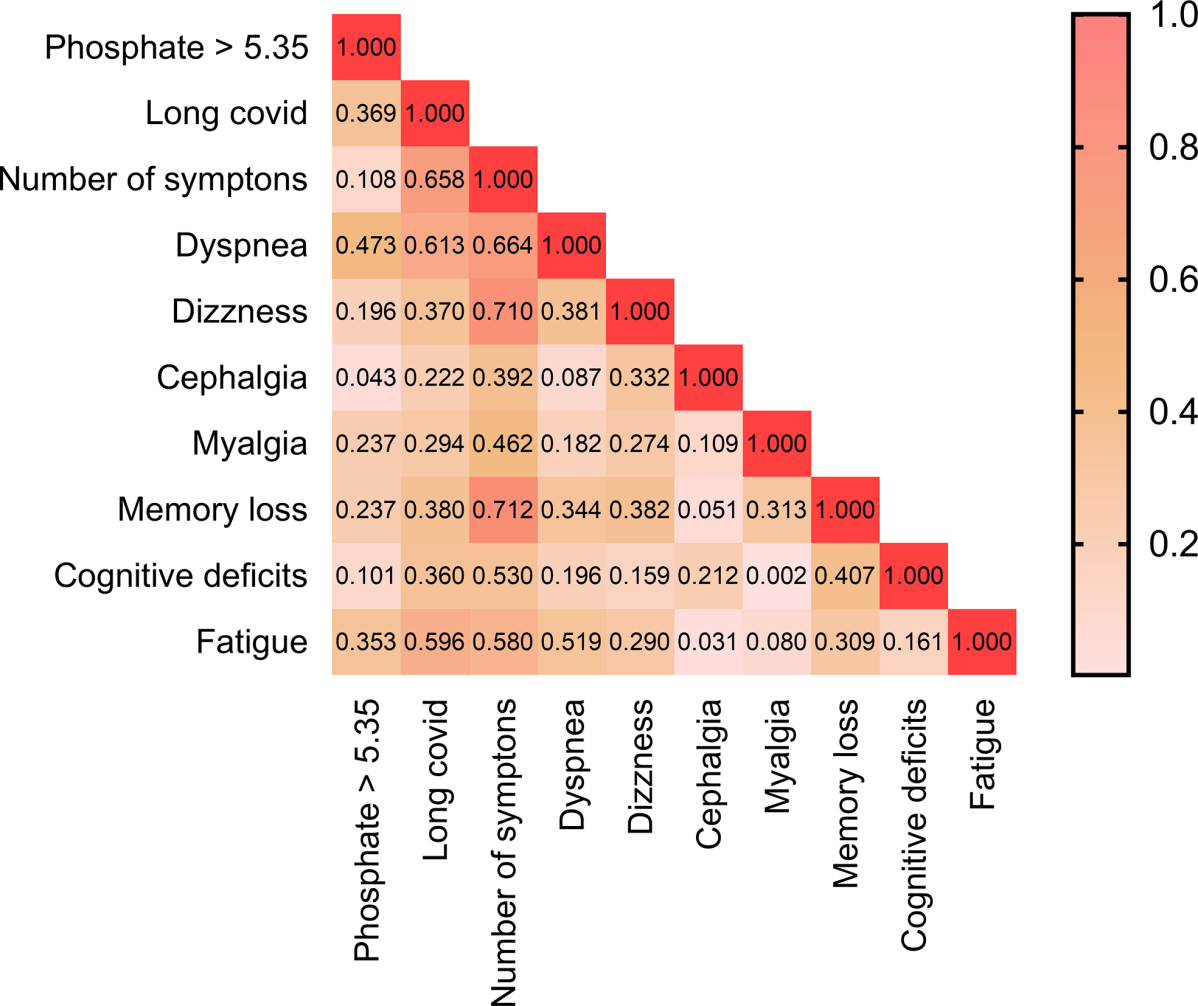
Heat map of the correlation matrix between phosphate ≥ 5.35 with each post COVID symptoms.

Patients with phosphate ≥ 5.35 mg/dL presented higher prevalence of long covid. Analyzing every symptom separately, it seems that subjects with higher values of phosphate are likely to present a higher prevalence of dyspnea and fatigue. See **Table 3** and **Figure 3**. Correlation matrix between these variables is presented on **Figure 2**.

**Table 3:** Long COVID according with phosphate concentrations.

Variables	Phosphate		X^2^	P value
	<5.35mg/dL	≥5.35 mg/dL		
Long covid	**17 (65.4)**	**21 (95.5)**	**6.533**	**0.011**
Dyspnea	**12 (46.2)**	**20 (90.9)**	**10.741**	**0.001**
Dizziness	8 (30.8)	11 (50)	1.843	0.175
Headache	5 (50)	5 (50)	0,088	0,766
Myalgia	6 (23.1)	10 (45.5)	2.685	0.101
Cognitive deficits	7 (26.9)	8 (36.4)	0.494	0.482
Fatigue	**11 (42.3)**	**17 (77.3)**	**5.994**	**0.014**

Data expressed by n (%). Chi-squared test was performed to compare groups. Bold values highlight statistical significances.

## Discussion

This study sought to characterize COVID-19 infection and long COVID symptoms from our cohort of 178 Brazilians HD patients, and to identify whether baseline characteristics should predict long COVID in this sample. We found that more than 85% of the COVID-19 infected patients presented a severe condition during the infection (hospitalized, needed supplementary O_2_, nursery or hospitalized in an intensity unit care). In our sample, the mortality rate over 11-month follow was relatively low (8.4%) when compared to the present literature (approximately 36%) (7). However, long COVID was highly prevalent in COVID-19 survivors representing more than 80% of all cases. A key finding of the study was the association of baseline phosphate and IL-10 with long COVID, demonstrating that higher levels of these two variables might be a risk factor for post-COVID complications. We suggested that patients with phosphate higher than 5.35 mg/dL may present an increased prevalence of long COVID, dyspnea, and fatigue. Although our sample size was not robust, these results may motivate further prospective and randomized trial to investigate deepest this relation between increased phosphate and long COVID.

In COVID-19-infected patients without CKD, higher levels of phosphate appear to be a protective factor against pulmonary damage, COVID severity, and mortality (21–24). Javdani et al. (25) suggests that hypophosphatemia is associated with severe lung injures, while increased serum phosphate may be associated with better CT scan of lung outcomes. Wang et al. (24) verified that hypophosphatemia at admission may be associated with increased mortality. Xue et al. (22) indicated that the severity of COVID-19 might be linked with lower phosphate levels. By this scenario, it would be rationale to infer those higher levels of phosphate would protect HD patients from long COVID since post-covid symptoms are likely related to COVID severity and organ damage (1, 4, 12). However, due to the decreased number of nephrons, CKD patients develop a compensate mechanism for phosphate balance characterized by the increase of fibroblast growth factor – 23 (FGF-23), parathormone, and decreased Vitamin D (Kuro-o, 2013; 26, 27). This mechanism precedes a hyperphosphatemia since the residual renal function are insufficient to excrete phosphate, leading to bone mineral disorders, inflammation, and interstitial fibrosis in HD patients (Kuro-o, 2013; 26, 27).

McGovern et al. (28) verified that higher serum phosphate concentrations (>1.5 mmol/L or >4.65 mg/dL) is associated with increased cardiovascular risk in CKD stages 3-5 (odds ratio = 2.34; 95% confidence interval = 1.64 – 3.32). This same study demonstrated that subjects without CKD could also present increased cardiovascular risk related to phosphate. Furthermore, even in normal ranges, greater serum phosphate is associated with vascular and valvular calcification in CKD patients (29). Noteworthy, HD patients hospitalized with COVID-19 seems to present hyperphosphatemia (30). Taken together, all this evidence underpins the results of the present study, suggesting higher serum phosphate as a possible predictors of persistence COVID symptoms. In this regard, further studies should address the impact of phosphate on COVID-19 prognosis, and long COVID prevalence among HD patients. Although our study did not present mechanistic insights, the role of phosphate in a worst prognosis of COVID patients with HD, might be related to an increased systemic inflammation (21, 31).

A striking feature of COVID-19 severity is the ‘citokine storm’ (32, 33). This phenomenon has been attributed as a clinical condition caused by an hyperactivation of immune system associated with several diseases (32). It is known that some cytokines are elevated on cytokine storm, including IL-10, TNF, and IL-6 (32, 33). Furthermore, these cytokines appear to be higher on patients infected with COVID-19 hospitalized in intensive care units (2). Although IL-10 acts as an anti-inflammatory cytokine inhibiting TNF and IL-6, it increased concentrations in cytokine storm might be likely due to insufficient counterregulatory action to the proinflammatory proteins (32). However, another hypothesis was made based on recent studies rising IL-10 as a possible proinflammatory cytokine in COVID-19 burden, but this concept must be further tested in detail (34, 35). These findings support the elevated concentrations of serum IL-10 in patients that developed long COVID presented in the present study. Suggesting that baseline IL-10 levels might be associated with post COVID symptoms. These results should encourage further researchers in identifying whether IL-10 levels could predict COVID-19 complications and long COVID.

This study presents several limitations that should be considered: first, this is a follow-up study which were not designed to analyze COVID-19 or long COVID *a priori*. However, considering the global burden of persistent COVID-19 in HD patients, this is a topic worth of investigation in the critical care of CKD patients. Second, the small sample size limits our inference in the general population with HD. Third, the lack of more time points of analysis limits our observation of the phenomenon. Finally, the lack of nutrition control limit our results, and we encourage further studies to control this variable. Nonetheless, to our knowledge, this is the first study that screened COVID and long COVID in HD patients and identified two possible targets (phosphate and IL10) of post-covid symptoms in this population.

In sum, there was a high prevalence of COVID-19 infection and long COVID in HD patients from the Brazilian trial ‘U1111-1237-8231’. Further studies should give a special look on serum phosphate and IL-10 levels in HD patients as possible targets of persistent COVID symptoms and severity.

## Data availability statement

The raw data supporting the conclusions of this article will be made available by the authors, without undue reservation.

## Ethics statement

The studies involving human participants were reviewed and approved by Ethic committee of Catholic University of Brasília. The patients/participants provided their written informed consent to participate in this study.

## Author contributions

HC, RN, LD, TA, AG, AR, RS, and TR: Conceptualization; HC, RN, LD, TA, AG, AR, RS, and TR: Data curation; HC: Formal analysis; CT-M and TR: Funding acquisition; HC, FH, RS, TA, DM-S, and TR: Investigation; Project administration; HC, DM-S, CT-M, CA, and TR: Roles/Writing - original draft; HC, DM-S, CA, and TR: Writing - review and editing. All authors contributed to the article and approved the submitted version.

## Funding

TSR, HLC, TBA, and LAD were supported by a grant provided by the Coordenação de Aperfeiçoamento de Pessoal de Nível Superior – Brazil (CAPES) – Finance Code 001 and National Council for Scientific and Technological Development (CNPq).

## Conflict of interest

The authors declare that the research was conducted in the absence of any commercial or financial relationships that could be construed as a potential conflict of interest.

## Publisher’s note

All claims expressed in this article are solely those of the authors and do not necessarily represent those of their affiliated organizations, or those of the publisher, the editors and the reviewers. Any product that may be evaluated in this article, or claim that may be made by its manufacturer, is not guaranteed or endorsed by the publisher.
